# Supplementary material for: Effects of Seasonal Anoxia on the Microbial Community Structure in Demosponges in a Marine Lake in Lough Hyne, Ireland
Source: mSphere. 2021 Feb 3;6(1):e00991-20. doi: 10.1128/mSphere.00991-20 (PMC7860989; doi:10.1128/mSphere.00991-20)
Supplement: TABLE S1 [file mSphere.00991-20-st001.docx]

| **Factor** | **df** | **F / Chi-squared** | **P** |
| --- | --- | --- | --- |
| **OTU1** -*E. sp2*  Kruskal-Wallis rank sum test |  |  |  |
| Oxygen | 2 | 1.87 | 0.393 |
| **OTU2** -*E. sp2*  Kruskal-Wallis rank sum test |  |  |  |
| Oxygen | 2 | 4.79 | 0.09 |
| **OTU3** -all data  Kruskal-Wallis rank sum test |  |  |  |
| Sample type | 3 | 30.0 | <0.001 |
| Wilcox pairwise test: Hs ≠ E ≠ S, W | | | |
| **OTU3** -*E.* sp.2  Kruskal-Wallis rank sum test |  |  |  |
| Oxygen | 2 | 0.542 | 0.763 |
| **OTU3** -*H. stellifera*  Kruskal-Wallis rank sum test |  |  |  |
| Oxygen | 2 | 0.156 | 0.925 |
| **OTU3** -Water  Kruskal-Wallis rank sum test |  |  |  |
| Oxygen | 2 | 7.64 | 0.0219 |
| Wilcox pairwise test: N ≠ H |  |  |  |
| **OTU3** -Sediment  Kruskal-Wallis rank sum test |  |  |  |
| Oxygen | 1 | 0 | 1 |
| **OTU7** -*E.* sp.2  Kruskal-Wallis rank sum test |  |  |  |
| Oxygen | 2 | 0.847 | 0.655 |
| **OTU7** -*H. Stellifera* ANOVA |  |  |  |
| Oxygen | 2 | 9.30 | 0.0145 |
| Residuals | 6 |  |  |
| Pairwise test: H<A | | | |
| **OTU6** -*E.* sp.2  ANOVA |  |  |  |
| Oxygen | 2 | 3.26 | 0.05 |
| Residuals | 24 |  |  |
| **OTU1075** -*E.* sp.2  ANOVA |  |  |  |
| Oxygen | 2 | 6.95 | 0.00416 |
| Residuals | 24 |  |  |
| Pairwise test: A>N,H | | | |
| **OTU17**- Sponge species  Kruskal-Wallis rank sum test |  |  |  |
| Sample type | 1 | 19.7 | <0.001 |
| Wilcox pairwise test: Hs ≠ E |  |  |  |
| **OTU17**-*E. s*p.2  Kruskal-Wallis rank sum test |  |  |  |
| Oxygen | 1 | 5.60 | 0.0608 |
| **OTU17**- *H. stellifera*  ANOVA |  |  |  |
| Oxygen | 2 | 0.939 | 0.442 |
| Residuals | 6 |  |  |
