# Supplementary material for: Effects of Seasonal Anoxia on the Microbial Community Structure in Demosponges in a Marine Lake in Lough Hyne, Ireland
Source: mSphere. 2021 Feb 3;6(1):e00991-20. doi: 10.1128/mSphere.00991-20 (PMC7860989; doi:10.1128/mSphere.00991-20)
Supplement: TEXT S1 [file mSphere.00991-20-s0001.docx]

**Supplementary material**

**Physical data:** To determine chlorophyll a (Chl *a*) and pheophytin concentrations, 50 mL of seawater from each depth was filtered through 25 mm diameter pre-combusted (450 °C for 4 h) GF/F filters. The filters were then placed in 2 mL Eppendorf® tubes wrapped in aluminium foil and frozen (-20 °C) until analysis. For analysis, the filters were transferred to 15 mL centrifuge tubes and acetone (8 mL, 90 %) was added. The samples were kept overnight (5 °C) before sonication (30 min) in a sonication bath and then centrifuged (3000 rpm at 6 °C for 5 min). Chlorophyll *a* was measured with a Turner TD-700 fluorometer (Turner Design, Sunnyvale, CA, USA). Samples were then acidified with 50 µL of 1 M HCl and pheophytin was measured. The fluorometer was calibrated using an extract from spinach and serial dilutions of a 4 mg L^-1^ stock standard, 90 % acetone was used as a blank. A solid-state secondary standard (SSS) was measured every ten samples. The SSS insert provides a very stable fluorescent signal and is used when measuring Chl *a* to check for fluorometer stability and sensitivity. The detection limit was 1 μg L^-1^.

**Microbial community sequencing:** For library preparations, up to 10 ng of DNA extracted from sponge, water and sediment samples was used as a template for PCR amplification of the 16S V4 rRNA gene amplicons, covering both *Bacteria* and *Archaea*. The PCR reaction (final volume 25 µl) was prepared with the following reagents: dNTPs (100 µM of each), MgSO4 (1.5 mM), Platinum Taq DNA polymerase HF (0.5 U/reaction), Platinum High Fidelity buffer (1X, Thermo Fisher Scientific, USA) and tailed primer mix (400 nM of each forward and reverse primer). The PCR program ran as follows: 95 °C for 2 min, 30 cycles of: 95 °C for 15 s, 55 °C for 15 s, and 72 °C for 50 s; and final elongation at 72 °C for 5 min. Duplicated PCR reactions were performed and pooled after PCR for each sample. Amplicon libraries were purified by a standard protocol for Agencourt Ampure XP Beads (Beckman Coulter, USA) with a bead ratio of 4:5. DNA was eluted in 25 µl nuclease free water (Qiagen, Germany). A Qubit dsDNA HS Assay kit (Thermo Fisher Scientific, USA) was used to measure DNA concentration. Validation of PCR product size and purity of sequencing libraries was carried out via Gel electrophoresis using Tapstation 220 and D1000/High sensitivity D1000 ScreenTapes (Agilent, USA). Sequencing libraries were prepared from the purified amplicon libraries using a second PCR containing PCRBIO HiFi buffer (1x), PCRBIO HiFI Polymerase (1 U/reaction) (PCR Biosystems, UK), adaptor mix (400 nM of each forward and reverse) and up to 10 ng of amplicon library template. The PCR settings were: 95°C for 2 min; 8 cycles of 95 °C for 20 s, 55 °C for 30 s, 72 °C for 60 s; and final elongation at 72 °C for 5 min. Sequencing libraries were purified again using Agencourt Ampure XP Beads (Beckman Coulter, USA) with a beat ratio 4:5, and DNA was eluted in 25 µl of nuclease-free water (Qiagen, Germany). Product size and purity of a subset of sequencing libraries was validated on a Tapstation 220 and D1000/High sensitivity D1000 ScreenTapes (Agilent, USA). A PhiX control library (>10%) was spiked in to overcome low complexity issues often observed with amplicon samples. After sequencing, Trimmomatic v. 0.32 [(148)](https://paperpile.com/c/KzF8J6/LnQSR) was used to quality trimm forward and reverse reads with settings: SLIDINGWINDOW: 5:3 and MINLEN: 250. The trimmed forward and reverse reads were merged using FLASH v. 1.2.7 [(149)](https://paperpile.com/c/KzF8J6/nFJvI) with settings: -m 10 -M 250. Trimmed reads were de-replicated and formatted for the use of the UPARSE workflow [(150)](https://paperpile.com/c/KzF8J6/BJfG2). Dereplicated reads were clustered by usearch v.7.0.1090 -cluster_otus command with default settings. The OTU abundances were estimated using -usearch_global command with -id 0.97 -maxaccepts 0 -maxrejects 0. Taxonomy was assigned using the RDP classifier [(151)](https://paperpile.com/c/KzF8J6/AYBzv) implemented in the paralles_assign_taxonomy_rdp.py script in QIIME [(152)](https://paperpile.com/c/KzF8J6/lhArU), using -confidence 0.8 and the SILVA database, release 132 [(153)](https://paperpile.com/c/KzF8J6/acPgS).

**Microbial communities:** The following information pertains to observations of sponge species that were not sampled under anoxia, i.e. either only in normoxia or hypoxia. *R.* *aculeta* contained four key OTUs: OTU1, OTU2 (*Gammaproteobacteria*), OTU3, and OTU10 (unassigned) in relative abundances of 11.8, 4.9, 9.7, and 11.8%, respectively (Fig. 7), all other OTUs present in *R.* *aculeata* had relative abundances lower than 4%. Similarly, *Hymeniacidon perlevis* had five OTUs that constituted 65% of its microbiome, including OTU47 (*Aphlaproteobacteria*, *Terasakiellaceae*) at 32.8%, OTU66 (*Alphaproteobacteria*, *Novosphingobiu*) at 12.1%, OTU271 (*Alphaproteobacteria*, *Terasakiellaceae*) at 7.3%, OTU8 (*Cyanobacteria*, *Synechococcus* CC990) at 6.5%, and OTU113 (*Gammaproteobacteria*) at 5.9% relative abundance. In contrast, *A. fucorum* contained only one key symbiont: OTU4 (*Gammaproteobacteria*), but it accounted for 46.2% of the microbiome by relative abundance. Lower relative abundances of OTU4 were observed in *Amphilectus* sp. (26.4%) along with OTU27 (also a *Gammaproteobacteria*) with 8% relative abundance. However, with the exception of *R. aculeta*, the aforementioned sponge species were collected at depths shallower than those exposed to seasonal anoxia (i.e. <24m). For the other 11 sponge species, there were only two key OTUs. The two key OTUs of *Eurypon* cf. *cinctum*, OTU145 (a *Nitrospira*) and OTU10 (Fig. 7). *Eurypon clavigerum* contained two *Gammaproteobacteria* OTUs as its main symbionts (OTU31 and 39), and the key symbionts of *Rhizaxinella* sp. were OTU3 (*Nitrosopumilus*-like) and OTU389 (candidatus *Nitrosopumilus*). In the remaining sponge species (i.e. excluding *Eurypon* cf. *cinctum, Eurypon clavigerum, Raspaciona* sp.*, Raspaciona* sp.1, and *Rhizaxinella* sp.), these two key OTUs consisted of one *Nitrosopumilus* (or *Nitrosopumilus*-like) and one *Gammaproteobacteria*. The specific combination of *Nitrosopumilus* (or *Nitrosopumilus*-like) and *Gammaproteobacteria* OTUs generally depended largely on the sponge species. As described above, *E.* sp.2 and *H. stellifera* microbiomes were primarily composed of the *Nitrosopumilus*-like OTU1 and the *Nitrosopumilus* OTU3, respectively, and *Gammaproteobacteria* OTUs 2 and 7, respectively. All *Endectyon* species, i.e. *Endectyon delaubenfelsi*, *Endectyon* sp.1 and *Endectyon* sp.2, had the *Nitrosopumilus* OTU1 and the *Gammaproteobacteria* OTU5 as key symbionts (Fig. 7).

References

148. Bolger AM, Lohse M, Usadel B. 2014. Trimmomatic: a flexible trimmer for Illumina sequence data. Bioinformatics 30:2114–2120.

149. Magoc T, Salzberg SL. 2011. FLASH: fast length adjustment of short reads to improve genome assemblies. Bioinformatics 27:2957–2963.

150. Edgar RC. 2013. UPARSE: highly accurate OTU sequences from microbial amplicon reads. Nat Methods 10:996–998.

151. Wang Q, Garrity GM, Tiedje JM, Cole JR. 2007. Naive Bayesian classifier for rapid assignment of rRNA sequences into the new bacterial taxonomy. Appl Environ Microbiol 73:5261–5267.

152. Caporaso JG, Kuczynski J, Stombaugh J, Bittinger K, Bushman FD, Costello EK, Fierer N, Peña AG, Goodrich JK, Gordon JI, Huttley GA, Kelley ST, Knights D, Koenig JE, Ley RE, Lozupone CA, McDonald D, Muegge BD, Pirrung M, Reeder J, Sevinsky JR, Turnbaugh PJ, Walters WA, Widmann J, Yatsunenko T, Zaneveld J, Knight R. 2010. QIIME allows analysis of high-throughput community sequencing data. Nat Methods 7:335–336[.](http://paperpile.com/b/KzF8J6/lhArU)

153. Quast C, Pruesse E, Yilmaz P, Gerken J, Schweer T, Yarza P, Peplies J, Glöckner FO. 2013. The SILVA ribosomal RNA gene database project: improved data processing and web-based tools. Nucleic Acids Res 41:D590–6[.](http://paperpile.com/b/KzF8J6/acPgS)
